# Atypical Focal Choroidal Excavation with Macular Hole in a Patient with Alagille Syndrome

**DOI:** 10.1155/2022/8136115

**Published:** 2022-09-05

**Authors:** Manami Misawa, Hironobu Tampo, Shinji Makino

**Affiliations:** Department of Ophthalmology, Jichi Medical University, Tochigi, Shimotsuke, Japan

## Abstract

This study is aimed at reporting a rare and unusual focal choroidal excavation with a macular hole in a patient with Alagille syndrome (AGS). A 21-year-old woman with an established early-life AGS diagnosis was referred to our hospital prior to liver transplantation. Examination revealed best-corrected visual acuity of 16/20 and 20/20 in the right and left eye, respectively. Slit-lamp examination was positive for posterior embryotoxon in both eyes. Fundoscopy revealed diffuse choroidal hypopigmentation with increased visibility of the choroidal blood vessels and circumferential chorioretinal atrophy in the mid-peripheral and peripheral retina in both eyes. A full-thickness macular hole with underlying focal choroidal excavation was observed in the right eye. Optical coherence tomography through the macula confirmed choroidal excavation with a full-thickness macular hole in the right eye. To our knowledge, this is the first report describing focal choroidal excavation with a macular hole in an AGS patient.

## 1. Introduction

Alagille syndrome (AGS) is clinically characterized by neonatal cholestatic jaundice with intrahepatic bile duct hypoplasia [1]. AGS is an autosomal dominant multiorgan disorder caused by aberrations in the Notch signaling pathway [2, 3], with an estimated prevalence of 1 in 30,000 to 70,000 live births [2, 3]. The majority (greater than 97%) of cases result from pathogenic variants in the Jagged canonical Notch ligand 1 (*JAG1*) gene on chromosome 20p12.18 with <1% of cases resulting from mutations in the *NOTCH2* gene (1p13) [2, 3]. Ocular findings include posterior embryotoxon, iris abnormalities, optic disc anomalies, and fundus changes [1–3].

Focal choroidal excavation (FCE) is a new disease entity of unknown origin first described by Jampol et al. [4] in 2006. FCE is defined as an area of concavity in the choroid, mostly in the macula and rarely in the extramacular region. Although initially considered congenital, an increasing number of cases have been reported in association with other retinochoroidal pathologies such as central serous choroidopathy, choroidal neovascularization, polypoidal choroidal vasculopathy, choroiditis, and choroidal tumors [5].

Herein, we report an atypical FCE with macular hole in an AGS patient.

## 2. Case Presentation

**Figure 1:**
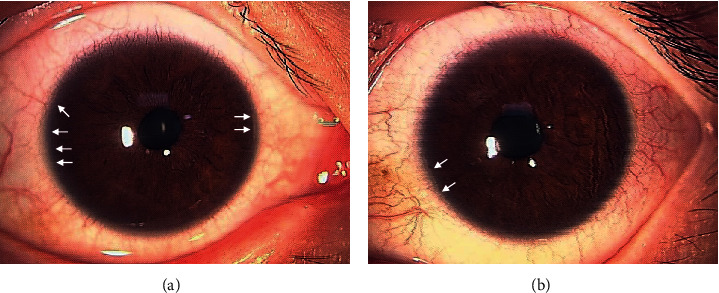
Slit-lamp photographs of the right (a) and left (b) eyes show posterior embryotoxon (arrows) and iris stromal atrophy.

**Figure 2:**
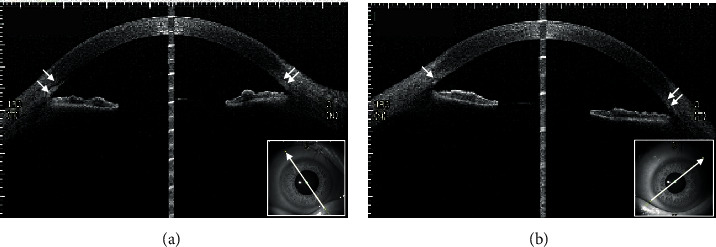
Anterior-segment optical coherence tomography images of the right (a) and left (b) eyes show posterior embryotoxon (arrows) and iris stromal atrophy.

**Figure 3:**
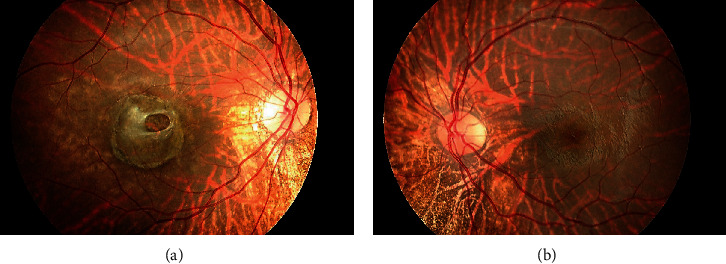
Fundus photographs of the right (a) and left (b) eyes. Note the diffuse choroidal hypopigmentation with increased visibility of the choroidal blood vessels. A full-thickness macular hole with underlying choroidal excavation observed in the right eye.

**Figure 4:**
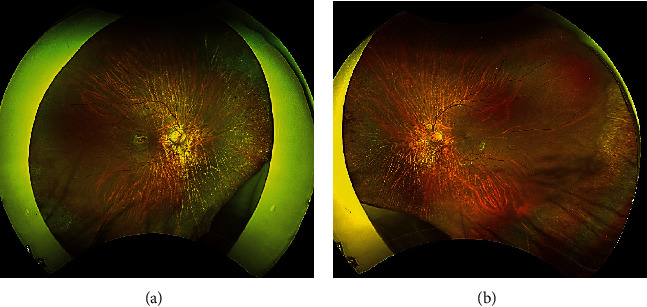
Right (a) and left (b) wide-field fundus photographs. Note a circumferential chorioretinal atrophy in the mid-peripheral and peripheral retina.

**Figure 5:**
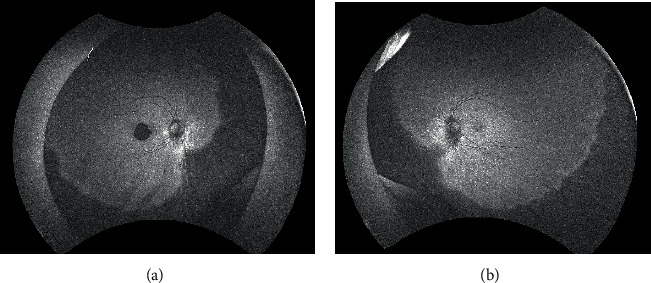
Right (a) and left (b) wide-field fundus autofluorescence images. Note the hypoautofluorescent areas corresponding with a circumferential chorioretinal atrophy in the mid-peripheral and peripheral retina. Autofluorescence loss is detected in the macular area in the right eye.

**Figure 6:**
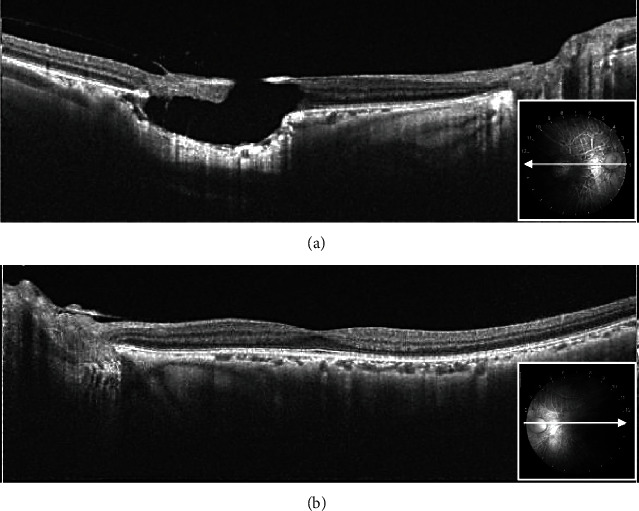
Right (a) and left (b) optical coherence tomography images. Note a full-thickness macular hole with underlying choroidal excavation in the right eye.

The patient was a 21-year-old woman diagnosed with AGS in early life. She was referred to the Jichi Medical University Hospital prior to liver transplantation. She had no problems with cognitive functioning. Her medical history was significant for systemic manifestations of AGS, including tetralogy of Fallot. On ophthalmic examination, the best-corrected visual acuity was 16/20 with −2.50D − 2.00D × 175° in the right eye and 20/20 with plane in the left. There was no abnormal ocular motility in either eye. Slit-lamp examination revealed posterior embryotoxon and iris stromal atrophy in both eyes (Figure 1). Anterior-segment optical coherence tomography (OCT) confirmed posterior embryotoxon and iris stromal atrophy (Figure 2). Fundoscopy revealed diffuse choroidal hypopigmentation with increased visibility of the choroidal blood vessels in both eyes (Figure 3). A full-thickness macular hole with underlying choroidal excavation was observed in the right eye (Figure 3(a)). Moreover, circumferential chorioretinal atrophy was detected in the mid-peripheral and peripheral retina (Figure 4). Fundus autofluorescence (FAF) imaging clearly revealed the hypoautofluorescent areas corresponding to circumferential chorioretinal atrophy (Figure 5). Further, loss of autofluorescence was detected in the macular area of the right eye (Figure 5(a)). An OCT scan through the macula demonstrated FCE with a full-thickness macular hole in the right eye (Figure 6(a)). The patient was scheduled to undergo liver transplantation.

## 3. Discussion

Here, we report unusual ocular findings in an AGS patient.

In 1999, Hingorani et al. [1] evaluated 22 AGS patients. The most common ocular abnormalities in AGS were posterior embryotoxon (95%), iris abnormalities (45%), diffuse fundus hypopigmentation (57%), speckling of the retinal pigment epithelium (33%), and optic disc anomalies (76%). Recently, da Palma et al. [2] examined 46 eyes in 23 AGS patients, identifying anterior-segment findings were present in 74% (17/23) of them. Posterior embryotoxon was the most common anterior chamber abnormality, identified in 70% (16/23) of patients. Iris stromal hypoplasia was present in five patients (22%). Posterior segment abnormalities were reported in 96% (22/23) of patients. Abnormalities of the optic disc were observed in 12 (52%) patients. Macular appearance varied from normal (22%) to diffuse atrophy, and 78% of all patients exhibited macular changes, including atrophy, pigmentation mottling, and granular changes. Peripheral chorioretinal abnormalities, reported in 22 (96%) patients, were the most frequent ocular findings in their series. This circumferential chorioretinal atrophy in the peripheral retina, assessed via wide-field FAF imaging, was first documented by Esmaili [6].

Interestingly, FCE with a full-thickness macular hole was observed in the present case. To our knowledge, this is the first report describing FCE with a macular hole in AGS patient. Few cases of FCE with a full-thickness macular hole have been reported in the literature. Hirawat et al. [7] reported FCE with a macular hole in a case of advanced retinitis pigmentosa. They speculated that inflammation due to retinitis pigmentosa could have caused disruption of the choroidal stroma, leading to atrophy and excavation. Although it is uncertain whether the development of FCE with a macular hole was consequential or coincidental, we speculate that in this patient, chorioretinal atrophy produced damage at the level of photoreceptors, the retinal pigment epithelium, and choriocapillaris, and then, the macular hole formed through the above mechanisms. The etiology of FCE remains unclear, and careful observation of fellow eyes with chorioretinal atrophy is required in such cases. Additional cases need to be examined to characterize these rare and unusual FCEs with macular holes in AGS. Ophthalmological evaluation with multimodal imaging should be pursued for all AGS patients.
